# Laparoscopic totally extraperitoneal hernia repair in patients with a history of previous abdominopelvic surgery

**DOI:** 10.1007/s13304-024-01810-w

**Published:** 2024-04-23

**Authors:** Romilly Hayward, Jacob J. Smith, Christos Kontovounisios, Shengyang Qiu, Oliver J. Warren

**Affiliations:** 1 Imperial College London School of Medicine, London, UK; 2 Chelsea and Westminster Hospital NHS Foundation Trust, 369 Fulham Road, London, SW10 9NH UK; 3 Department of Surgery and Cancer, Imperial College London, London, UK

**Keywords:** Hernia, Laparoscopic, Total extraperitoneal, Inguinal, Groin, Previous surgery, Abdominopelvic

## Abstract

A retrospective cohort study of patients undergoing laparoscopic inguinal hernia repair compared short- and long-term outcomes between individuals with or without history of previous abdominopelvic surgery, aiming to determine the feasibility of totally extraperitoneal (TEP) repair within this population. All patients who underwent elective TEP inguinal hernia repair by one consultant surgeon across three London hospitals from January 2017 to May 2023 were retrospectively analysed to assess perioperative outcomes. Two hundred sixty-two patients were identified, of whom two hundred forty-three (93%) underwent laparoscopic TEP repair. The most frequent complications were haematoma (6.2%) and seroma (4.1%). Recurrence occurred in four cases (1.6% of operations, 1.1% of hernias). One hundred eighty-four patients (76%) underwent day-case surgery. There were no mesh infections or explanations, vascular or visceral injuries, port-site hernias, damage to testicle, or persisting numbness. There were no requirements for blood transfusion, returns to theatre, or readmissions within 30 days. There was one conversion to open and one death within 60 days of surgery. Eighty-three (34%) had a history of previous AP surgery. There was no significant difference in perioperative outcomes between the AP and non-AP arms. This finding carried true for subgroup analysis of 44 patients whose AP surgical history did not include previous inguinal hernia repair and for those undergoing repair of recurrent hernia. In expert hands, laparoscopic TEP repair is associated with excellent outcomes and low rates of long-term complications, and thus should be considered as standard for patients regardless of a history of AP surgery.

## Background

Inguinal hernia repairs are amongst the most frequent abdominal surgeries in the UK with over 60,000 performed within NHS England each year [1]. Despite extensive collective experience and knowledge within the field, there is not yet consensus on the optimum surgical approach.

Laparoscopic repair is increasingly preferred over open repair, associated with reduced recovery time and lower rates of chronic pain and numbness [2]. There are two standardised laparoscopic approaches, transabdominal pre-peritoneal (TAPP) and totally extraperitoneal (TEP) [3]. TEP repair is minimally invasive and unlike TAPP, the repair does not require violation of the peritoneal cavity, reducing the risk of injury to intra-peritoneal organs [4]. However, both TEP and TAPP are associated with steep learning curves and considered more technically challenging than open procedures [2]. Moreover, many surgeons view certain patient factors such as a history of abdominopelvic (AP) surgery as relative contraindications to TEP repair; hence, the approach is not always considered feasible [5, 6]. Previous AP surgery is widely considered a relative contraindication to laparoscopic repair owing to the expectation that this approach would prove more complex in the presence of intra-abdominal adhesions [7].

A retrospective cohort study of patients undergoing laparoscopic inguinal hernia repair was conducted to compare short- and long-term outcomes between individuals with or without history of previous AP surgery, aiming to determine the feasibility of TEP repair within this population.

## Methods

All patients who underwent elective TEP inguinal hernia repair by one consultant surgeon across three London hospitals from January 2017 to May 2023 were retrospectively analysed to assess perioperative outcomes.

### Surgical technique

A standard three-port multi-trocar TEP repair was performed under general anaesthesia. Blunt dissection using a balloon trocar and subsequent carbon dioxide insufflation using a Hasson’s cannula through a 1-cm subumbilical incision was performed to create the initial working space. Reduction of the hernia sac was achieved through two midline working ports, one 2–3 cm above the pubic symphysis and one in the midway between the other two ports. Polypropylene mesh (Bard 3D Max™) was introduced through the infraumbilical port, placed over the defect and secured without fixation.

### Data collection

Data were retrieved from preoperative clinic notes, operative notes and letters to General Practitioners. Standard follow-up occurred at 2 and 6 weeks. All patient identifiable data were removed, and data were collated in a dedicated electronic database.

Outcome measures included conversion rates, early complications (<30 days) (need for reintervention, haematoma, seroma, superficial or mesh infection, vascular or visceral injury, damage to testicle, port-site hernia) and late complications (>30 days) (persisting numbness (>2 months), chronic pain (>2 months), recurrence).

Groups were categorised for comparison based on history of previous AP surgery with subgroup analysis excluding previous inguinal hernia repair. Further subcategorisation was conducted based on hernia characteristics (primary versus recurrent; unilateral versus bilateral).

### Statistical analysis

Data were analysed using IBM SPSS Statistics programme version 28.0.0.0 (190). Continuous parametric data are reported as mean (standard deviation), continuous non-parametric data as median (interquartile range) and categorical data as frequency (percentage). Groups were compared using unpaired two-tailed *t* tests (continuous parametric), Mann–Whitney rank sum tests (continuous non-parametric) and Chi-square tests (categorical). *P* values <0.05 were considered significant.

## Results

Two hundred sixty-two patients underwent elective inguinal hernia repair between January 2017 and May 2023. Laparoscopic TEP repair with Bard 3D Max™ mesh was offered as standard, and was used in 243 cases (93%). Nineteen patients (7%) underwent Lichtenstein open repair with prolene mesh, the most common indications for open repair being previous laparoscopic repair, previous lower midline major surgery, inguinoscrotal hernia, and patient preference.

### Outcomes in laparoscopic totally extraperitoneal repair

One hundred fifty TEP repairs (62%) were unilateral and ninety-three (32%) were bilateral; thus, a total of three hundred fifty-six hernias were repaired laparoscopically. Median (IQR) length of follow-up was 2.18 (1.07–4.01) years. Two hundred seven patients were male (85%) with a median age of 60 years (IQR: 47–71). Eighty-three patients (34%) had a history of previous AP surgery and twenty-one (9%) presented for repair of a recurrent hernia.

Complication rates are summarised in Table 1. The most frequent complications were haematoma (6.2%) and seroma (4.1%), all cases of which resolved spontaneously without the need for further reintervention. Recurrence occurred in four cases (1.6% of operations, 1.1% of hernias). One hundred eighty-four patients (76%) underwent day-case surgery.

**Table 1:** Surgical outcomes following elective laparoscopic totally extraperitoneal inguinal hernia repair

	TEP repair (*n* = 243)
Early complication (<30 days)	26 (10.7%)
Late complication (>30 days)	6 (2.5%)
Superficial infection	4 (1.6%)
Haematoma	15 (6.2%)
Seroma	10 (4.1%)
Chronic pain	2 (0.8%)
Recurrence	4 (1.6%)
Overnight stay	59 (24.3%)

Values are number (proportion)

There were no mesh infections or explanations, vascular or visceral injuries, port-site hernias, damage to testicle or persisting numbness. There were no requirements for blood transfusion, returns to theatre or readmissions within 30 days. There was one conversion to open and one death within 60 days of surgery.

### TEP repair outcomes for patients with and without history of abdominopelvic surgery

Of the 243 patients who underwent laparoscopic TEP repair, 83 (34%) had a history of previous AP surgery.

Patient demographics and hernia characteristics were similar between groups (Table 2).

**Table 2:** Patient demographics and hernia characteristics for patients with or without a history of abdominopelvic surgery who underwent elective laparoscopic inguinal hernia repair

	Non-AP (*n* = 160)	AP (*n* = 83)	*P* value
Male (*n*)	140 (88%)	67 (81%)	0.158^†^
Age (years)	58 (45–71)	62 (54–71)	0.131^§^
Bilateral repair (*n*)	65 (41%)	28 (34%)	0.295^†^
Length of follow-up (years)	2.17 (1.05–4.05)	2.18 (1.13–3.56)	0.985^§^

Values are median (IQR) or number (proportion)

*AP* previous abdominopelvic surgery history, *non-AP* no abdominopelvic surgery history

*P* values calculated using ^†^ Chi-squared test; ^§^ Mann–Whitney *U* test

There was no significant difference in perioperative outcomes between the AP and non-AP arms (Table 3).

**Table 3:** Comparison of outcomes for patients with or without a history of abdominopelvic surgery following laparoscopic inguinal hernia repair

	Non-AP (*n* = 160)	AP (*n* = 83)	*P* value^†^
Early complication (<30 days)	18 (11.3%)	8 (9.6%)	0.700
Late complication (>30 days)	3 (1.9%)	3 (3.6%)	0.407
Superficial infection	4 (2.5%)	0 (0%)	0.146
Haematoma	8 (5.0%)	7 (8.4%)	0.292
Seroma	7 (4.4%)	3 (3.6%)	0.777
Chronic pain	1 (0.6%)	1 (1.2%)	0.635
Recurrence	2 (1.3%)	2 (2.4%)	0.500
Overnight stay	36 (22.5%)	23 (27.7%)	0.369

Values are number (proportion)

*AP* previous abdominopelvic surgery history, *non-AP* no abdominopelvic surgery history

^†^ Calculated using Chi-squared test

### AP subgroup analysis—excluding previous inguinal repair

Thirty-nine patients whose AP surgical history included previous inguinal hernia repair were excluded from subgroup analysis. The AP arm, thus, consisted of 44 patients, and the non-AP arm of 160.

Thirteen patients had a history of multiple previous AP surgeries. The most frequent types of previous AP surgery included: appendectomy (*n* = 21), urological procedures (*n* = 13, of which the most common was prostatectomy, *n* = 4), gynaecological procedures (*n* = 10, of which the most common was caesarean section, *n* = 6), and bowel resection (*n* = 4).

There was a greater proportion of male patients in the AP arm. Other patient demographics and hernia characteristics were similar between groups (Table 4).

**Table 4:** Patient demographics and hernia characteristics for patients with or without a history of abdominopelvic surgery who underwent elective laparoscopic inguinal hernia repair

	Non-AP (*n* = 160)	AP (*n* = 44)	*P* value
Male (*n*)	140 (88%)	33 (75%)	0.041^†,*^
Age (years)	58 (45–71)	62 (53–73)	0.258^§^
Bilateral repair (*n*)	65 (41%)	18 (41%)	0.973^†^
Length of follow-up (years)	2.17 (1.05–4.05)	2.30 (1.29–3.55)	0.854^§^

Values are median (IQR) or number (proportion)

*AP* previous abdominopelvic surgery history (excluding inguinal hernia repair), *non-AP* no abdominopelvic surgery history

^*^ *P* < 0.05

*P* values calculated using ^†^ Chi-squared test; ^§^ Mann–Whitney *U* test

There was no significant difference in perioperative outcomes between the AP and non-AP arms (Table 5).

**Table 5:** Comparison of outcomes for patients with or without a history of abdominopelvic surgery following laparoscopic inguinal hernia repair

	Non-AP (*n* = 160)	AP (*n* = 44)	*P* value^†^
Early complication (<30 days)	18 (11.3%)	4 (9.1%)	0.683
Late complication (>30 days)	3 (1.9%)	2 (4.5%)	0.310
Superficial infection	4 (2.5%)	0 (0%)	0.289
Haematoma	8 (5.0%)	4 (9.1%)	0.307
Seroma	7 (4.4%)	2 (4.5%)	0.961
Chronic pain	1 (0.6%)	1 (2.3%)	0.326
Recurrence	2 (1.3%)	1 (2.3%)	0.681
Overnight stay	36 (22.5%)	11 (25.0%)	0.727

Values are number (proportion)

*AP* previous abdominopelvic surgery history (excluding inguinal hernia repair), *non-AP* = no abdominopelvic surgery history

^†^ Calculated using Chi-squared test

### Outcomes in bilateral versus unilateral TEP repair

One hundred fifty TEP repairs (59.5%) were unilateral and ninety-three (40.5%) were bilateral. Length of follow-up was greater in the bilateral arm (*P* = 0.018) (Table 6).

**Table 6:** Patient demographics and hernia characteristics for patients undergoing laparoscopic repair of unilateral versus bilateral inguinal hernia

	Unilateral (*n* = 150)	Bilateral (*n* = 93)	*P* value
Male	127 (85%)	80 (86%)	0.773^†^
Age (years)	61 (48.3–74.0)	59 (45.0–69.0)	0.274^§^
Recurrent hernia	13 (8.7%)	8 (8.6%)	0.986^†^
Abdominopelvic surgical history	55 (36.7%)	28 (30.1%)	0.295^†^
Length of follow-up (years)	1.91 (1.03–3.68)	2.67 (1.57–4.38)	0.018*^,§^

Values are median (IQR) or number (proportion)

^*^ *P* < 0.05

*P* values calculated using ^†^ Chi-squared test; ^§^ Mann–Whitney *U* test

There was no significant difference in overall early complication rates or any late complication rates for unilateral versus bilateral repair. Patients who underwent bilateral repair had higher incidence of seroma (7.5% versus 2%, *P* = 0.035) and overnight stay (31.2% versus 20%, *P* = 0.048) (Table 7).

**Table 7:** Comparison of outcomes for patients undergoing laparoscopic repair of unilateral versus bilateral inguinal hernia

	Unilateral (*n* = 150)	Bilateral (*n* = 93)	*P* value^†^
Early complication (<30 days)	13 (8.7%)	13 (14.0%)	0.193
Late complication (>30 days)	4 (2.7%)	2 (2.2%)	0.801
Superficial infection	2 (1.3%)	2 (1.3%)	0.627
Haematoma	9 (6.0%)	6 (6.5%)	0.887
Seroma	3 (2.0%)	7 (7.5%)	0.035*
Chronic pain	0 (0%)	2 (2.2%)	0.071
Recurrence	4 (2.7%)	0 (0%)	0.112
Overnight stay	30 (20.0%)	29 (31.2%)	0.048*

Values are number (proportion)

^†^ Calculated using Chi-squared test

### Outcomes following TEP repair of primary versus recurrent hernias

Two hundred twenty-two TEP repairs (91%) were for primary hernias and twenty-one (9%) were for recurrent hernias. Patient demographics and hernia characteristics were similar between groups (Table 8).

**Table 8:** Patient demographics and hernia characteristics for patients undergoing elective laparoscopic repair of primary versus recurrent inguinal hernia

	Primary (*n* = 222)	Recurrent (*n* = 21)	*P* value
Male	190 (86%)	17 (81%)	0.568^†^
Age (years)	60.0 (46.0–71.0)	61.0 (57.0–73.0)	0.380^§^
Bilateral hernia	85 (38%)	8 (38%)	0.986^†^
Length of follow-up (years)	2.19 (1.07–3.85)	2.05 (1.07–4.38)	0.607^§^

Values are median (IQR) or number (proportion)

*P* values calculated using ^†^ Chi-squared test; ^§^ Mann–Whitney *U* test

There was no significant difference in perioperative outcomes between the primary and recurrent arms (Table 9).

**Table 9:** Comparison of outcomes for patients undergoing laparoscopic repair of primary versus recurrent inguinal hernia

	Primary (*n* = 222)	Recurrent (*n* = 21)	*P* value^†^
Early complication (<30 days)	22 (9.9%)	4 (19.0%)	0.195
Late complication (>30 days)	6 (2.7%)	0 (0%)	0.446
Superficial infection	4 (1.8%)	0 (0%)	0.535
Haematoma	12 (5.4%)	3 (14.3%)	0.106
Seroma	9 (4.1%)	1 (4.8%)	0.876
Chronic pain	2 (0.9%)	0 (0%)	0.662
Recurrence	4 (1.8%)	0 (0%)	0.535
Overnight stay	51 (23.0%)	8 (38.1%)	0.122

Values are number (proportion)

^†^ Calculated using Chi-squared test

## Discussion

This dataset provides real-world insight into outcomes after inguinal hernia repair performed by a surgeon experienced in laparoscopic techniques. Excellent outcomes are reported following TEP repair, with low complication and recurrence rates. These findings carry true for patients with a history of previous AP surgery, suggesting that for experienced surgeons, such history should not be a contraindication to laparoscopic repair. Overall short- and long-term complication rates were similar for bilateral versus unilateral repair, lending favour to the opportunistic repair of incidental asymptomatic hernias discovered during the primary repair.

### TEP repair for patients with previous abdominopelvic surgery history

Patient characteristics and medical history are key considerations when deciding on surgical approach, especially history of previous pelvic or lower abdominal surgery. Previous AP surgery is widely considered a relative contraindication to laparoscopic repair owing to the expectation that this approach would prove more complex in the presence of intra-abdominal adhesions [7]. This study found no increased risk of complications for patients with a history of AP surgery or for those presenting with a recurrent hernia following laparoscopic repair, with no conversions to open repair in either subgroup. The European Hernia Society recommends recurrent inguinal hernias should be repaired using the opposite approach to the previous surgery [8]. For example, after previous open repair via an anterior approach, laparoscopic repair (TEP/TAPP) via the posterior route is preferred. Although the Lichtenstein technique is recommended following prior laparoscopic repair, European Association of Endoscopic Surgery guidelines consider laparoscopic repair following previous TEP/TAPP, a complex situation that ought to be elected by surgeons with experience in minimally invasive surgery [9, 10]. To fully assess the feasibility of laparoscopic TEP repair in the hands of an experienced surgeon, subgroup analysis was performed for patients with a history of AP surgery that excluded previous repair of inguinal hernia. This analysis showed no significant difference in post-operative outcomes between the AP and non-AP arms, further indicating the safety and feasibility of laparoscopic TEP repair these patients.

Whilst this data is from a single surgeon, findings nonetheless demonstrate that, regardless of past surgical history, laparoscopic TEP repair is an excellent option for most patients when performed by an experienced surgeon. This is supported by previous research demonstrating similar outcomes following TEP repair for patients with or without history of previous lower abdominal surgery [6, 7]. Patients should, therefore, be informed and consented with consideration of their surgeon’s preferred approach and relevant expertise. Moreover, given the strong correlation between a surgeon’s experience and improved post-operative outcomes [11–13], alongside reduced recovery time and long-term complication rates observed for laparoscopic versus open repair [14, 15], increased emphasis on training surgeons in laparoscopic techniques could hold benefit in improving patient outcomes and reducing costs and healthcare burden.

### Outcomes in bilateral versus unilateral TEP repair

Although incidence of seroma and overnight stay were greater following bilateral repair, overall short-term complications were similar between groups and there was no significant difference in any long-term complications. Overnight stay was often a decision based on convenience, taking into account the time of surgery and distance travelled by patients to return home, in particular for those who were elderly, comorbid, or who lived alone. Subsequently, the finding of increased overnight stay in the bilateral group may have been confounded by these factors. Prevalence of asymptomatic contralateral inguinal hernia in those with unilateral inguinal hernia is around 22%, with 30% eventually becoming symptomatic [16, 17]. With no increased risk of long-term complications or recurrence observed for patients undergoing bilateral versus unilateral repair, this dataset lends favour to the practise of identification and opportunistic repair of contralateral asymptomatic hernias during repair of the primary to reduce future morbidity.

### Determining optimal surgical approach

Different surgical approaches for inguinal hernia repair have been extensively reviewed and compared to determine the optimal method, with outcomes shown to vary depending on numerous factors. Guidelines determined by the HerniaSurge group in 2018 reflected this, recommending that the choice of surgical approach be tailored to ‘the surgeon’s expertise, patient- and hernia-related characteristics, and local/national resources’, with further emphasis on the idea that ‘one single standard technique for all hernias does not exist’ [8].

The recommendation to consider the operating surgeon’s preference, training, and capabilities when deciding on surgical approach was strongly supported by the results of this study where, in the hands of a surgeon experienced in laparoscopic techniques, this approach was associated with excellent outcomes. In the literature, complication rates for TEP range from 1.3 to 50.3% (median 12.5%). A review of these complications with regard to the Clavien–Dindo classification determined 22% to be clinically relevant in the long term (Grade ≥ III) [18]. This study reports 19% of complications as long term, giving an overall long-term complication rate of just 2.5% with recurrence rate 1.6%.

As surgeons gain experience with each respective approach, outcomes tend to improve [8, 11, 13]. Prospectively, low-volume surgeons (25–30 surgeries per year) have markedly higher recurrence rates compared to high-volume surgeons [12]. The trend holds true for those experienced in a single approach, with retrospective analysis of 2410 TEP repairs conducted by the Mayo Clinic demonstrating a correlation between higher annual surgeon volume and decreased perioperative complications, overnight stays and recurrence rates [19]. It is, therefore, recommended that inguinal hernia surgery be conducted by an experienced high-volume surgeon specialising in their preferred technique [20].

This has led to difficulty in designing valid randomised controlled trials to compare surgical approaches; hence, alternative methods of evaluating clinical outcomes are required. Moreover, increasing utilisation of robotic surgery and the emergence of robotic TAPP repair have necessitated the creation of a framework to quickly assess clinical outcomes and learning curves associated with novel surgical techniques. This study demonstrates the routine evaluation of a surgeon’s practise is a suitable, low-cost and highly feasible approach to outcome analysis, in high-volume centres. This approach provides up-to-date, real-world clinical data, allowing surgeons to track their progress and respective learning curves over time, and providing patients with accurate and applicable information to facilitate informed consent.

### Limitations

The findings of this study were restricted to the practise of one experienced London-based surgeon, reducing the generalisability of results. It would be important to apply this model to other high-volume hernia surgeons and centres to test its validity. Furthermore, the retrospective design meant certain demographic data including BMI, smoking status and activity levels were incomplete, which may have confounded results. Future studies should include detailed demographic data, alongside more structured follow-up to screen for recurrence and long-term complications. The association between history of laparoscopic and open repair in patients presenting with recurrence may also be considered to further analyse this correlation. This will be well supported by the launch of the British Hernia Society Registry similar to the Danish Hernia Database which has proved indispensable in clinical research and improvement of patient outcomes [21, 22].

## Conclusion

In expert hands, laparoscopic TEP repair is associated with excellent outcomes and low rates of long-term complications, and thus should be considered as standard for patients regardless of a history of AP surgery or hernia recurrence. Given no increased risk of long-term complications or recurrence following bilateral repair, surgeons may consent patients undergoing unilateral repair for potential concomitant repair of incidental contralateral hernia to reduce future morbidity.

## Data Availability

All data underlying the results are included in this article or are available from the corresponding author on reasonable request.
